# Systemic Signature of the Lung Response to Respiratory Syncytial Virus Infection

**DOI:** 10.1371/journal.pone.0021461

**Published:** 2011-06-24

**Authors:** Jeroen L. A. Pennings, Annemieke Schuurhof, Hennie M. Hodemaekers, Annemarie Buisman, Lia C. G. H. de Rond, Myra N. Widjojoatmodjo, Willem Luytjes, Jan L. L. Kimpen, Louis Bont, Riny Janssen

**Affiliations:** 1 Laboratory for Health Protection Research, National Institute for Public Health and the Environment, Bilthoven, The Netherlands; 2 Centre for Infectious Disease Control Netherlands, National Institute for Public Health and the Environment, Bilthoven, The Netherlands; 3 Department of Pediatrics, Wilhelmina Children's Hospital, University Medical Center Utrecht, Utrecht, The Netherlands; 4 Netherlands Vaccine Institute, Bilthoven, The Netherlands; South Texas Veterans Health Care System, United States of America

## Abstract

Respiratory Syncytial Virus is a frequent cause of severe bronchiolitis in children. To improve our understanding of systemic host responses to RSV, we compared BALB/c mouse gene expression responses at day 1, 2, and 5 during primary RSV infection in lung, bronchial lymph nodes, and blood. We identified a set of 53 interferon-associated and innate immunity genes that give correlated responses in all three murine tissues. Additionally, we identified blood gene signatures that are indicative of acute infection, secondary immune response, and vaccine-enhanced disease, respectively. Eosinophil-associated ribonucleases were characteristic for the vaccine-enhanced disease blood signature. These results indicate that it may be possible to distinguish protective and unfavorable patient lung responses via blood diagnostics.

## Introduction

Most children become infected with respiratory syncytial virus (RSV) within the first two years of their life. Infants infected with RSV show disease courses that range from uneventful upper respiratory tract symptoms to severe bronchiolitis requiring mechanical ventilation [1], [2]. Although several risk factors for severe disease are known [3]–[5], including host genetic factors, many aspects of host susceptibility are still poorly understood. In previous studies we used gene expression analysis to characterize the lung response upon primary and secondary RSV infection in detail in a murine model [6], [7], and showed a robust innate immune response at day 1 and 2 after RSV infection. The predominant signature of the primary infection was the rapid innate immune response characterized by the induction of large numbers of chemokines and interferon (IFN) regulated genes. In mice protected against RSV by primary infection that were subsequently challenged with RSV, this early innate response was almost identical to the response in primary infection, but was followed by a rapid decrease in time concomitant with viral disappearance. In contrast, mice with formalin-inactivated RSV (FI-RSV) vaccine-enhanced disease displayed a prolonged innate immune lung response with characteristics of Th2 polarization.

In recent years, there have been several studies that applied blood transcriptomics for lung inflammatory disease diagnostic purposes [8]–[12]. However, eventual clinical implementation of blood transcriptomics relies on the extent to which its results represent molecular lung pathology during the disease course. In the case of RSV, insight into the correlation between the lung and blood response during primary RSV bronchiolitis may guide the development of methods for blood-based identification and monitoring of host responses as a proxy for the disease process that is ongoing in the lung. Additionally, a more detailed insight into which aspects of lung responses are represented at the systemic (i.e. blood) level, is required to improve our understanding of host responses to RSV infection in children. In this study, we describe the comparison between gene expression responses in lung, bronchial lymph nodes, and whole-blood in a mouse model upon primary and secondary RSV infection. We identified an innate immunity-associated gene set that gives correlated responses in these three tissues and characterized the systemic signature of the local lung infection. Next we focused on systemic signatures that can distinguish mice with a distinct presentation of infection. We identified gene blood gene expression signatures indicative of acute infection, secondary immune response, and vaccine-enhanced disease, respectively.

## Materials and Methods

### Ethics statement

This study was agreed upon by the Animal Experimentation Ethical Committee of our institute under permit number 200700171. Animal handling in this study was carried out in accordance with relevant Dutch national legislation, including the 1997 Dutch Act on Animal Experimentation.

### Animal experiment

All experimental procedures have been described before [7] and were performed in accordance with national and institute guidelines. Briefly, BALB/c mice of 6–10 weeks of age (n = 6 per group) were infected intranasally with 10^6^ pfu RSV type A2 or mock 7 weeks before challenge, or intramuscularly injected with 10^7^ pfu FI-RSV or FI-mock 7 and 3 weeks before challenge. At day 0, mice were challenged intranasally with 10^6^ pfu RSV. Mice were sacrificed unchallenged or at day 1, 2, or 5. The right lung and bronchial lymph nodes were collected in RNAlater (Applied Biosystems) and used for RNA isolation according to described methods [6], [7]. Blood was collected in a K_2_-EDTA blood collection tube (Terumo). Subsequently, 0.5 ml blood was added to 1.3 ml RNAlater, stored overnight at 4°C, and until RNA isolation stored at −20°C. Total RNA was isolated with the Mouse RiboPure -Blood RNA isolation kit, followed by DNase treatment with the DNA-free kit, and after ethanol precipitation depleted for globin mRNA with the GLOBINclear -Mouse/Rat kit (all blood RNA kits from Applied Biosytems). RNA concentrations were measured using a NanoDrop Spectrophotometer (Thermo Scientific) and RNA quality was determined using a Bioanalyzer (Agilent). Prior to further microarray analyses, RSV infection was verified by realtime PCR on RNA isolated from the lung as described [7].

### Microarray analysis

RNA amplification and labeling were carried out with the Amino Allyl MessageAmp II aRNA kit (Applied Biosystems) according to the manufacturers' instruction, using 1.5 µg of total RNA as starting material. RNA samples from individual mice (n = 4–5 per group) were labeled and hybridized against a common reference containing a labeled amino allyl-modified aRNA pool of all samples isolated. Microarray slides containing 22,680 oligos from the Sigma-Compugen Mouse oligonucleotide library (and appropriate controls) were spotted at the Microarray Department of the University of Amsterdam. Arrays were scanned at two wavelengths using a ScanArray 4000XL microarray scanner (Perkin-Elmer). Following microarray scanning, median Cy3 and Cy5 signal intensities per spot were determined using Array Vision software (Imaging Research). Quality control was performed on raw data by means of visual inspection of the scanned images, as well as a check on the scatter and MA plots.

To control for possible batch effects during RNA isolation, labeling, or hybridization, experiments were carried out according to a randomized block design, so that samples for each biological group were assigned evenly across handling days. As part of the QC it was verified by PCA and ANOVA that no significant batch effects could be found and (technical) variance due to possible batch effects was smaller than (biological) variance within or between experimental groups.

All raw and normalized microarray data are MIAME compliant and were deposited at the ArrayExpress website (http://www.ebi.ac.uk/arrayexpress/) under accession numbers E-TABM-826 and E-TABM-1094.

### Data analysis

Data analysis was performed in accordance with methods we used previously [6], [7]. Briefly, raw signal data from oligo-containing spots were normalized in R using a three-step approach of natural log-tranformation, quantile normalization of all scans, and correcting the sample spot signal for differences in the corresponding reference spot signal between arrays. Differentially expressed genes were identified with ANOVA, using stringency criteria of p<0.001 and a >2-fold induction (i.e. up-regulated by challenge) unless indicated otherwise. This p-value corresponded to a False Discovery Rate of <5% for all three tissues. Gene expression patterns were visualized in R and GeneMaths XT (Applied Maths). Functional annotation and Gene Ontology (GO) term overrepresentation were assessed using the DAVID functional annotation tool (http://david.abcc.ncifcrf.gov/) [13]. Overrepresentation of tissue-specific or literature-based functional gene sets was determined in R using an in-house developed algorithm based on the DAVID methodology, using gene sets extracted from data at BioGPS website (http://biogps.gnf.org) [14], [15] as well as other relevant literature [16]-[19]. Additional functional annotation was performed by textmining using Anni 2.1 (http://www.biosemantics.org/anni/) [20]. Lists of regulated genes were compared by calculating the cosine correlation, which for binary lists equals R = N_a&b_/√(N_a_*N_b_).

Enrichment for GO or other functional terms was further visualized as a molecular concepts analysis by showing functional terms and regulated genes per tissue as nodes and significant (p<0.01) enrichment as edges. To compare expression changes across days and tissues, a modular representation [17] was made of gene expression changes. Modules included were relevant tissue responses or disease course signatures as described in this study, functional terms based on GO or Uniprot, and immune cell type-based gene sets based on literature [15], [17], [18]. Median expression changes per module were calculated and then visualized in GeneMaths XT.

## Results

In our previous study, gene expression in the lungs of mice upon primary and secondary RSV infection was compared. In the current study, the response in the bronchial lymph nodes and blood from the same mice was determined. To identify the systemic signature of the lung response, these blood and lymph-node responses were first determined and compared to the previously described lung response [7] of these mice.

In bronchial lymph nodes of primary RSV-infected mice, 192 genes were induced. Functional annotation showed that a large percentage of genes were involved in the immune and inflammatory response. Interferon signaling genes were up-regulated, as well as genes associated with NK cells or cytotoxic lymphocyte function (such as granzyme A and B). Additionally, at day 5, up-regulation of immunoglobulin genes as well as several hematological developmental genes was found (such as *Pou2af1*, *Shh*, *Xbp1*). This indicates plasma cell maturation and the onset of antibody production at this time point.

**Figure 1:**
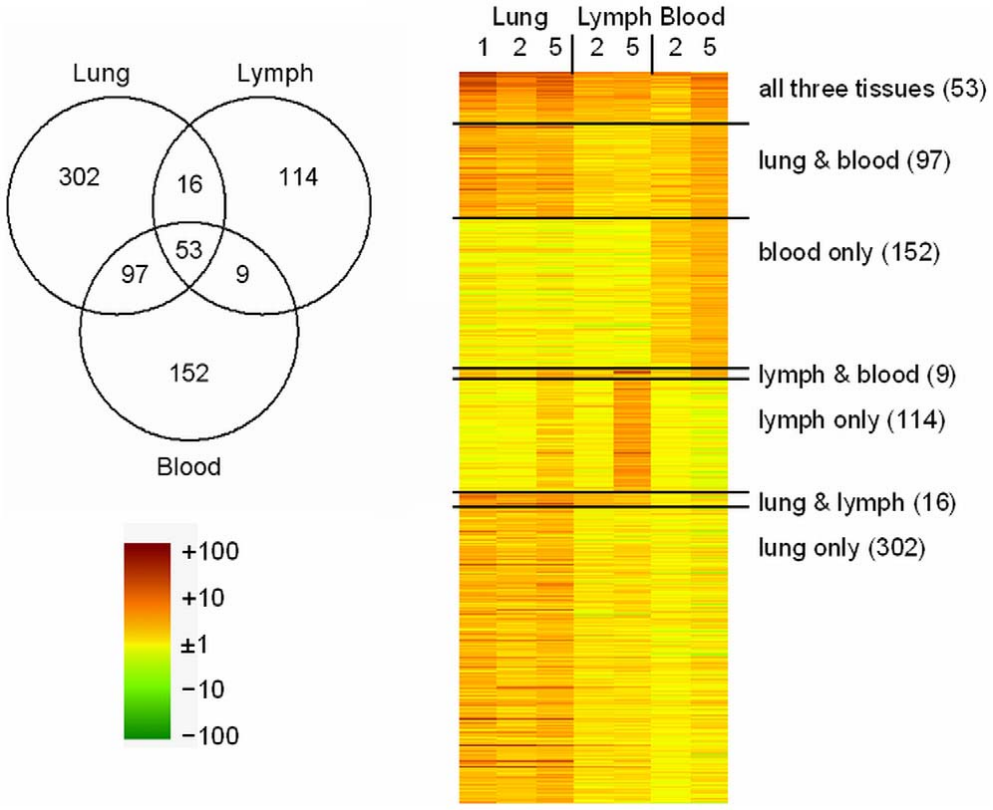
Comparison between lung, lymph node, and blood responses. The Venn diagram indicates the number of genes regulated in common or specific for each of the three tissues across any of the days examined. The heatmap indicates gene expression changes relative to the median of the unchallenged group on days 1, 2, and 5.

Blood responses showed significant up-regulation for 311 genes. Among the regulated genes, there was significant enrichment for immune and inflammatory response genes, especially interferon responsive genes. These responses were more pronounced at day 5, when levels of regulation were comparable to those found in lung (Figure 1). Additionally, we found up-regulation of a number of (neutrophil) granulocyte-associated genes (such as *Cd177*, *Fpr1*, *Mmp8*, *Ncf1*, *Sell*), indicating changes in the blood cell population numbers.

**Figure 2:**
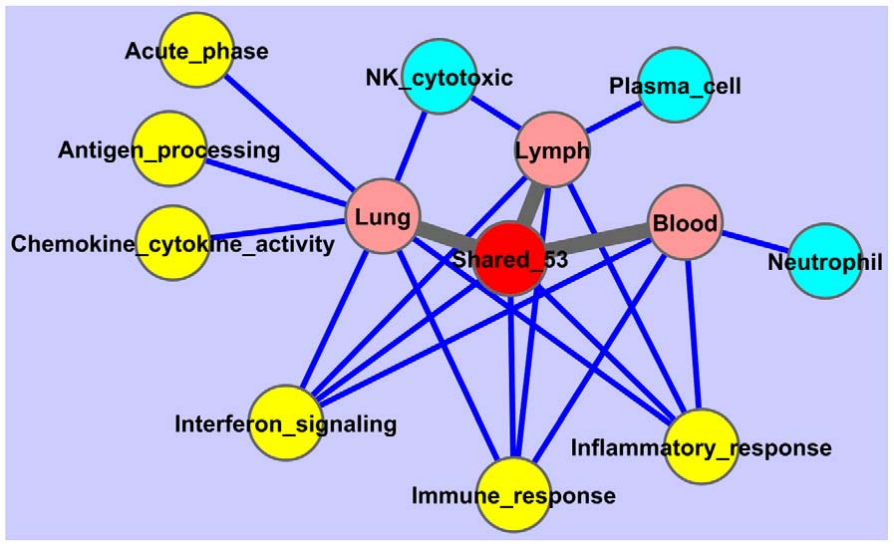
Functional enrichment for lung, lymph node, and blood responses. Nodes represent tissue responses (pink), shared primary response (red), GO/UniProt functional terms (yellow) and immune cell type (light blue). Edges represent significant (p<0.01) enrichment (dark blue) or a subset relation (gray).

The previously described lung signature induced upon primary RSV infection encompassed 468 genes (p<0.001, minimally two-fold induction). As reported previously [7], functional annotation showed a significant enrichment for immune and inflammatory response genes, and several involved mechanisms including chemokine or cytokine activity, interferon responses, and antigen processing. These genes were all highly expressed at days 1, 2, and 5 (Figure 1). Enrichment for biological process or cell type-associated genes among genes regulated in lung, lymph node, or blood is visualized in Figure 2. As can be seen from the Figure, immune response, inflammatory response, and IFN-signaling are enriched in all three tissues.

**Figure 3:**
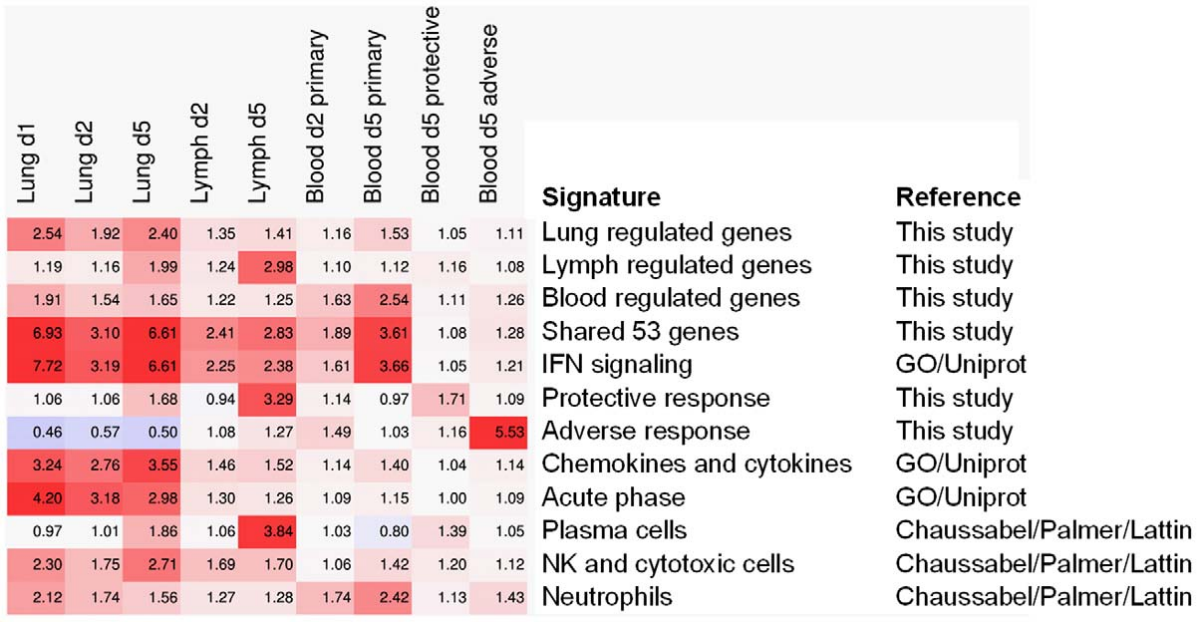
Module comparison of expression responses. Figures indicate the median response per module. Background color indicates the extent of up- (red) or down-regulation.

When induced responses between the three tissues were compared across any of the days examined, the largest overlap was seen between lung and blood. With 150 overlapping up-regulated genes (R = 0.39), this was larger than the overlap between lung and lymph nodes (69 genes, R = 0.23) or between lymph nodes and blood (62 genes, R = 0.25) (Figure 1). A group of 53 genes was identified which was induced in all three tissues in response to RSV infection (Figure 1, Table 1). Gene expression patterns between these genes were in good agreement, with pairwise correlations based on these 53 genes always being larger than 0.8. As described for the individual tissues, interferon signaling was the predominant process found among the functional annotations for these genes (Figure 2). Furthermore, other immune-related functions such as inflammation and antigen processing were found among these genes, as were cell cycle and apoptosis (Figure 2). One chemokine (*Cxcl9*) was found among these shared genes. A modular representation of gene expression changes (Figure 3) shows that the median response for the shared genes followed the time course kinetics in the three individual tissues. The extent of regulation was most pronounced for lung, followed by blood and lymph nodes (Figure 3).

**Table 1:** Gene expression changes upon RSV infection in mice for the shared set of 53 genes in lung, lymph node, and blood on days 1, 2, and 5.

Gene symbol	Synonym	Lung d1	Lung d2	Lung d5	Lymph d2	Lymph d5	Blood d2 primary*	Blood d5 primary	Blood d5 protective*	Blood d5 adverse*	Primary function
*5830458K16Rik*	*Rtp4*	10.70	6.50	5.64	4.39	3.51	4.95	8.09	1.39	2.57	unknown
*Ccnd1*		2.62	1.66	1.80	2.17	1.49	3.36	1.66	0.94	0.88	cell-cycle
*Clec7a*		1.09	1.95	2.29	1.35	2.32	2.96	4.02	1.07	1.43	inflammation
*Cxcl9*		19.27	4.36	15.28	3.29	3.14	1.31	2.18	1.01	1.28	chemokine
*Daxx*		5.83	2.44	3.23	2.00	1.60	1.31	2.10	1.05	0.95	apoptosis
*G1p2*		45.72	15.08	22.79	7.03	5.32	5.52	1.59	0.62	0.49	interferon
*Gata3*		1.91	1.46	2.26	1.59	2.18	1.06	2.27	1.23	1.04	inflammation
*Gbp1*		5.06	1.91	6.61	2.20	3.03	1.55	8.30	1.19	1.33	interferon
*Gbp2*		6.93	2.72	8.37	2.05	3.71	1.66	9.47	1.08	1.17	interferon
*Gbp4*		9.61	3.65	7.84	1.89	2.94	1.33	5.24	1.33	1.46	interferon
*Gvin1*		2.27	1.69	2.58	2.14	2.19	1.42	2.64	1.05	1.30	interferon
*Gzmb*		4.99	3.10	12.01	3.76	4.93	1.59	2.52	1.59	1.53	inflammation
*H28*	*Ifi44L*	9.95	6.50	7.48	3.03	3.04	2.08	4.21	0.98	1.13	antigen processing
*H2-M3*		2.78	2.40	2.66	2.01	1.63	1.45	2.80	1.07	1.51	antigen processing
*Herc5*		8.30	3.17	3.70	2.04	1.88	1.95	2.29	1.13	1.03	antigen processing
*Ifi1*		11.02	5.07	11.50	3.67	4.69	2.32	8.11	1.09	1.35	interferon
*Ifi202b*		19.89	9.47	10.52	2.75	3.21	1.78	4.46	1.23	1.39	interferon
*Ifi203*		8.02	3.68	4.74	2.26	1.78	1.20	2.34	1.06	1.00	interferon
*Ifi204*		18.43	10.24	12.93	2.36	2.52	1.60	3.60	1.11	1.40	interferon
*Ifi44*		29.77	15.43	12.00	4.26	3.16	3.40	5.32	1.22	1.15	interferon
*Ifi47*		5.86	2.31	4.96	2.32	2.35	1.36	5.52	0.84	1.23	interferon
*Ifit1*		30.41	9.17	10.59	4.53	3.99	8.96	6.13	1.01	0.92	interferon
*Ifit3*		22.57	8.33	7.71	3.99	3.26	3.69	5.70	1.05	1.28	interferon
*Ifitm3*		3.45	3.03	2.86	2.22	1.73	5.74	4.38	1.10	1.79	interferon
*Igtp*		8.77	3.47	12.06	2.47	4.50	2.40	3.66	1.15	1.23	interferon
*Iigp2*		7.99	3.08	10.02	2.37	3.75	1.98	3.68	1.06	1.15	interferon
*Il18bp*		10.52	5.03	11.27	1.82	2.52	1.58	7.55	1.02	1.42	inflammation
*Irf7*		28.88	12.41	12.18	9.17	4.43	8.97	3.37	0.57	0.62	interferon
*Isgf3g*	*Irf9*	2.56	2.18	1.63	2.22	1.79	1.61	2.14	1.00	1.07	interferon
*Ly6a*		3.23	2.88	4.61	5.10	5.33	1.93	3.97	1.01	1.23	various functions
*Ly6c*		4.17	2.50	3.47	2.97	3.65	2.81	8.95	1.08	2.06	various functions
*Ly6f*		3.83	2.37	2.75	1.91	2.41	2.56	7.48	1.09	1.41	various functions
*Mpa2*	*Gbp4*	4.91	1.77	8.13	2.58	3.42	1.12	4.26	0.89	1.02	inflammation
*Ms4a11*		3.04	2.14	2.68	2.05	1.75	1.22	2.71	1.10	1.75	various functions
*Ms4a6d*		15.59	11.16	12.27	4.40	2.83	2.00	6.87	1.37	2.54	various functions
*Oas1a*		12.65	6.42	7.80	2.41	2.19	2.44	3.95	1.09	1.64	DNA/RNA
*Oasl2*		10.46	6.23	6.53	1.88	2.13	5.41	10.38	1.10	1.25	DNA/RNA
*Parp9*		5.05	3.27	3.59	2.25	2.28	1.89	3.24	1.01	1.04	various functions
*Pkib*		1.58	1.73	2.12	2.40	2.33	1.27	2.87	1.20	1.16	various functions
*Plac8*		10.16	6.85	9.35	4.45	4.03	2.90	10.18	1.28	3.10	unknown
*Rbm3*		1.83	1.58	2.02	1.94	2.60	1.38	2.23	1.19	1.18	DNA/RNA
*Samhd1*		3.15	1.57	3.80	2.42	3.05	1.40	5.27	1.30	1.59	inflammation
*Serpina3g*		9.29	6.06	21.83	3.06	5.42	1.46	13.41	0.99	1.39	apoptosis
*Six2*		2.93	1.93	3.26	2.02	2.67	1.19	2.77	1.01	1.16	DNA/RNA
*Slfn1*		3.75	2.90	3.56	2.65	2.13	2.24	3.63	1.18	1.38	cell-cycle
*Slfn3*		7.72	3.05	4.63	2.78	2.11	2.27	4.18	0.68	0.86	cell-cycle
*Slfn4*		24.47	13.55	8.11	4.44	3.41	7.14	12.16	1.09	1.85	cell-cycle
*Socs1*		4.04	1.69	6.85	1.90	2.38	1.02	2.74	0.98	1.08	inflammation
*Socs3*		2.70	2.25	3.54	2.23	1.77	1.63	2.45	1.17	1.32	inflammation
*Stat1*		6.46	3.62	6.86	2.62	3.88	1.32	5.06	1.18	1.56	interferon
*Tgtp*		16.07	5.28	19.28	3.67	4.53	3.68	5.14	1.05	1.03	inflammation
*Xmr*		3.72	2.67	3.75	2.53	2.44	2.10	6.98	1.13	1.77	cell-cycle
*Zbp1*		12.64	6.80	10.96	3.67	3.67	1.88	4.69	1.04	1.11	DNA/RNA

Blood data are indicated as follows: primary, primary RSV infection response; protective, secondary RSV infection response; adverse, FI-RSV vaccine-enhanced response. Data for lung and lymph node are all responses to primary RSV infection. Values are given as ratios between infected and control.

Interferon signaling and an inflammatory response were also the main processes among the 97 genes induced in lung and blood but not in lymph nodes, indicating that responses in lung and blood strongly overlap with respect to these processes. In contrast, regulation of acute phase markers (*e.g. Saa1*, *Saa2*, *Saa3*, *Lcn2*) was almost exclusively seen in the lung, as were most chemokines and cytokines (Figure 2, Figure 3). Functional annotations seen only in blood were (neutrophil) granulocyte-associated genes, whereas immunoglobulin and plasma cell-associated genes are characteristic for the genes that were only regulated in bronchial lymph nodes (Figure 2, Figure 3).

For each of the three tissues, we also determined which genes were down-regulated. In blood, we found only eight down-regulated genes (p<0.001, minimally two-fold decrease). None of these eight genes showed overlap with either the 241 genes down-regulated in lung, or the 29 genes down-regulated in bronchial lymph nodes. Therefore, in contrast to the up-regulated responses, no shared down-regulated response could be identified.

During secondary infection of mice with RSV, the response was much weaker than during primary infection, consistent with rapid antibody-mediated viral clearance. Interestingly, especially the shared IFN signature was not up-regulated in blood at day 5 (median ratio to control 1.08) indicating that this is clearly a marker for acute infection (median ratio 3.61) (Figure 3). In mice with vaccine-enhanced disease, this response was still detectable at day 5 (median ratio 1.28), supporting the hypothesis that vaccine-enhanced disease is mediated by an enhanced innate immunity response in the presence of viral clearance. This is also reflected in lung responses reported previously [7] as well as the expression of a neutrophil-associated module (Figure 3).

After establishing that the gene expression response in the blood of mice with primary RSV infection is, at least in part, a reflection of the response that is raised at the site of infection, i.e. the lung, we focused on blood signatures that can distinguish mice with a distinct presentation of infection. Such signatures would allow determination of the extent of lung infection through blood diagnostics. For this purpose we determined the blood signatures of either a protective (secondary) response or an adverse (FI-RSV enhanced) response. As these responses were less clear-cut than the acute response, we allowed for more genes meeting the stringency criteria by making these slightly less stringent (p<0.001, induction > 1.41-fold (i.e. a half log2-step)). We found five such markers for the secondary response (*IgG1*, *Cxcr3*, *Eaf2*, *3110040K02Rik, 4921506M07Rik*), and four for the vaccine-enhanced disease (*Ear1*, *Ear2*, *Ear3*, *Ear6*). The former of these sets showed specific induction in mice with secondary infection (median ratio  =  1.71 versus 0.97 in primary infection or 1.09 in vaccine-enhanced disease) (Figure 3) whereas the effects in the latter were specific for the vaccine-enhanced disease (median ratio 5.53 versus 1.03 in primary and 1.16 in secondary response) (Table 2, Figure 3). Remarkably, the identified markers for vaccine-enhanced disease were all eosinophil-associated ribonucleases, which is in agreement with the Th2-polarized response found in the lung.

**Table 2:** Gene expression changes upon RSV infection in mice for primary infection, secondary infection, and vaccine-enhanced signatures in blood on day 5.

Signature	Blood d5 primary	Blood d5 protective	Blood d5 adverse
Primary infection			
Shared 53 genes			
(see Table 1)			
Median	3.61	1.08	1.28
All IFN-regulated			
Median	3.66	1.05	1.21
Secondary infection			
*IgG1*	1.37	12.7	9.66
*Cxcr3*	0.96	1.71	1.09
*Eaf2*	0.87	1.51	1.04
*3110040K02Rik*	0.90	1.99	1.05
*4921506M07Rik*	0.97	1.62	1.12
Median	0.97	1.71	1.09
Vaccine-enhanced			
*Ear1*	1.03	1.28	7.63
*Ear2*	1.02	1.31	7.31
*Ear3*	1.10	1.06	3.03
*Ear6*	0.73	0.80	3.76
Median	1.03	1.16	5.53

Values are given as ratios between infected and control.

## Discussion

The aims of our studies were two-fold. First, we wished to establish if the systemic gene expression response to infection, which can be measured in the blood, is a reflection of the local response that is ongoing in the lung. Our studies show that over 30% of the genes induced in mouse lungs upon RSV infection were also regulated in the blood transcriptome. A shared set of biologically relevant genes was identified. Second, we used blood transcriptomics to distinguish mice with a distinct presentation of infection. Indeed, favorable and unfavorable responses could be distinguished: induction of IFN regulated genes was indicative of acute infection and absent during secondary infection whereas induction of eosinophil-associated ribonuclease (Ear) genes was indicative of vaccine-enhanced disease (Table 2, Figure 3).

The systemic signature identified in our study is in line with a study in humans by Fjaerli et al. [8], who used microarray and quantitative real-time PCR to study blood gene expression responses to RSV. Their study reported significant differential expression for 30 genes, including several interferon responsive genes, in infants hospitalized with RSV bronchiolitis that did not require artificial ventilation. Their controls were infants from the same birth-cohort that never suffered from RSV bronchiolitis and did not suffer from RSV infection at the time of blood sampling. Although our studies were performed in mice, it is interesting to note that *Ifi27*, which they reported as the most strongly up-regulated gene, was also found significantly up-regulated in our study (6.96-fold). Additionally, we found significant up-regulation for five other genes reported as up-regulated by Fjaerli et al. (*Ifi44*, *H28*/*Ifi44L*, *G1p2*, *Hp*, *Clu*), the first three of these being IFN regulated. These genes were most prominently regulated in our groups of mice with primary RSV infection and probably reflect a state of active viral replication in the lung. In mice that clear the infection this response was less prominent. The fact that our data in mice display similarity with responses described in infants suggest a similar systemic signature of acute RSV infection in mice and humans.

Interferon-regulated responses appear to be part of the innate response to viral and to some extent to bacterial pathogens. Zaas et al. [9] reported whole-blood gene expression signatures in response to RSV, rhinovirus, and influenza, in a cohort of healthy adult volunteers. These healthy volunteers all showed mild symptoms upon RSV infection. Viral infection signatures allowed to accurately discriminate viral infections from bacterial infections (*Streptococcus pneumoniae*, *Staphylococcus aureus*, and *Escherichia coli*) or uninfected controls, but were mutually very comparable. Of the genes reported in at least one of their viral top-30 signatures, twelve were also regulated in our blood data (*Cxcl10*, *Gbp1*, *H28*/*Ifi44l*, *Herc5*, *Ifi27*, *Ifi44*, *Ifit1*, *Ifit2*, *Ifit3*, *Ly6e*, *Socs1*, *Stat1*), again including mostly interferon responsive genes. Of these, *Gbp1* was only present in the RSV signature. Ramilo et al. [10] described classifiers to distinguish between pediatric patients with acute influenza, *S. pneumoniae*, *S. aureus*, or *E. coli* infection. Their influenza classifier genes showed mostly IFN regulated genes, several of which (*Ifi44*, *G1p2*, *Oas1*, *Zbp1*) showed overlap with our signature. By contrast, in their classifiers to distinguish bacterial infections from influenza or between different bacterial infections, practically no IFN regulated genes were found and only *Stat1* overlapped with our blood signature. Predominantly IFN regulated signatures were also reported by Thach et al. [11] in basic military trainees with adenoviral febrile respiratory illness. Additionally, IFN responses were found in peripheral blood of cynomolgus macaques after smallpox infection [21]. Taken together, these studies show the role of interferon responsive genes as part of a conserved response in blood during acute viral infection or other febrile illness and that the blood response in humans and in murine models displays extensive similarities.

The finding that interferon signaling is involved in the gene expression response in lung and blood, but also to some extent in lymph nodes, is in agreement with previous studies that found a pivotal role for the innate immune system in RSV clearing and susceptibility to severe disease [6], [7], [22]. Indeed, for several shared genes whose primary function is annotated to other pathways (Table 1), there is evidence for interferon-dependent regulation, or such regulation can be inferred through interferon-dependent regulation of closely related homologs. Examples of this include two oligoadenylate synthetase genes (*Oas1a*, *Oasl2*) [23], and three slafen family members (*Slf1*, *Slf3*, *Slf4*) [24]. Altogether, for 38 genes such an association could be established. Moreover, when promoter sequences of the remaining genes were examined (using the oPOSSUM software at www.cisreg.ca/oPOSSUM [25]), two IRF1 transcription factor binding sites were found upstream of the *Pkib* gene, suggesting that even more genes might be regulated by interferon signaling.

Besides other viral response signatures, the systemic IFN signature reported in this study showed significant overlap with blood signatures found in patients with systemic lupus erytromatosus (SLE) [17], and LPS response in mice [26]. By contrast, this overlap was not (or not significantly) found in patients with juvenile idiopathic arthritis [27] or with an ionizing radiation [28] or chemotherapy [26] response. Recently, Berry et al. [12] reported an IFN-inducible whole-blood gene expression signature that distinguishes active tuberculosis from latent tuberculosis or healthy controls. Furthermore, in the study by Ramilo et al. [10] about one third of patients with bacterial infections displayed elevated expression levels of IFN regulated genes, although the authors indicate it is unclear if these might have an undiagnosed or preceding viral infection. Thus, although IFN responses seem to be an essential part of the antiviral response both in humans and in murine models, these studies indicate that it is not specific (nor clearly aspecific) for viral infections. Confirmation of RSV or other viral infections in clinical diagnostics therefore needs to come from assays specific for detection of individual viruses. Conversely, host blood signatures indicative of severe RSV infection may not be specific for RSV infection and may be a marker of disease severity, irrespective of the causative virus.

In the study by Zaas et al. [9], expression of IFN blood signatures distinguished between mild symptomatic and asymptomatic cases, and a similar IFN signature was also found in hospitalized children described by Fjaerli et al. [8]. Because these two studies used a different study design and age groups and both a relatively homogenous case and control groups, it is not possible to determine to what extent IFN signatures correlated with disease severity within or between these groups. By way of comparison, expression of IFN signatures has been found to correlate with disease severity in SLE [17] and tuberculosis [12]. Also, in our study, after secondary infection with RSV the magnitude of the shared IFN signature response differs from that of the primary response, and additionally differs between a protective and an adverse response. This suggests our blood signature might be useful in a blood-based diagnostic or even prognostic application. Moreover, a signature consisting of all IFN-dependent genes regulated in blood (51 out of 311 genes, 38 overlapping with the shared response) also shows differential expression between primary, protective, and adverse responses (Table 2). However, both our mouse study and human literature evidence to date are insufficient to determine to what extent blood signatures are indicative of RSV disease severity in children. Further studies will therefore be needed with suitable cohort groups that also allow addressing issues such as sensitivity towards other kinds of blood-detectable disease or stress responses.

Our results suggest that blood-based mRNA analysis can be used to recognize an unfavorable secondary Th2 response in case of vaccine-enhanced disease. *Ear1/2/3/6* expression was a typical characteristic of the blood transcriptome during vaccine-enhanced disease following RSV challenge in mice who previously received the formaline-inactivated RSV vaccine. These genes are associated with eosinophils and allergic asthma, and are involved in host response against respiratory viral pathogens [29], [30]. This also applies to their corresponding human orthologs *RNASE2* and *RNASE3* [29], [30]. If the findings of our study can be translated to humans, this may provide a powerful tool to assess safety of any future RSV vaccine tested in humans. In particular, induction of a Th2-biased response through the vaccine may be revealed at early stage of vaccine development.

Taken together comparison of our murine data with human literature data indicates that it may be possible to deduce patient lung responses via the blood compartment. Distinct profiles of blood biomarker genes are indicative of acute inflammation, protection, and vaccine-enhanced disease in mouse RSV models. Although for each of these signatures, specificity and relevance for disease severity warrants further studies, this opens up possibilities for studies aimed at predicting vaccine safety. Further translation of such non-invasive testing methods to children may allow better disease monitoring and treatment.
